# Response mechanisms of *Saccharomyces cerevisiae* to the stress factors present in lignocellulose hydrolysate and strategies for constructing robust strains

**DOI:** 10.1186/s13068-022-02127-9

**Published:** 2022-03-15

**Authors:** Bo Li, Nan Liu, Xuebing Zhao

**Affiliations:** 1 Key Laboratory of Industrial Biocatalysis, Ministry of Education, Tsinghua University, Beijing, 100084 China; 2 Institute of Applied Chemistry, Department of Chemical Engineering, Tsinghua University, Beijing, 100084 China

**Keywords:** Lignocellulosic biomass, Stress factor, Stress response, Target genes, Robust strain construction

## Abstract

Bioconversion of lignocellulosic biomass to biofuels such as bioethanol and high value-added products has attracted great interest in recent decades due to the carbon neutral nature of biomass feedstock. However, there are still many key technical difficulties for the industrial application of biomass bioconversion processes. One of the challenges associated with the microorganism *Saccharomyces cerevisiae* that is usually used for bioethanol production refers to the inhibition of the yeast by various stress factors. These inhibitive effects seriously restrict the growth and fermentation performance of the strains, resulting in reduced bioethanol production efficiency. Therefore, improving the stress response ability of the strains is of great significance for industrial production of bioethanol. In this article, the response mechanisms of *S. cerevisiae* to various hydrolysate-derived stress factors including organic acids, furan aldehydes, and phenolic compounds have been reviewed. Organic acids mainly stimulate cells to induce intracellular acidification, furan aldehydes mainly break the intracellular redox balance, and phenolic compounds have a greater effect on membrane homeostasis. These damages lead to inadequate intracellular energy supply and dysregulation of transcription and translation processes, and then activate a series of stress responses. The regulation mechanisms of *S. cerevisiae* in response to these stress factors are discussed with regard to the cell wall/membrane, energy, amino acids, transcriptional and translational, and redox regulation. The reported key target genes and transcription factors that contribute to the improvement of the strain performance are summarized. Furthermore, the genetic engineering strategies of constructing multilevel defense and eliminating stress effects are discussed in order to provide technical strategies for robust strain construction. It is recommended that robust *S. cerevisiae* can be constructed with the intervention of metabolic regulation based on the specific stress responses. Rational design with multilevel gene control and intensification of key enzymes can provide good strategies for construction of robust strains.

**Graphical Abstract:**

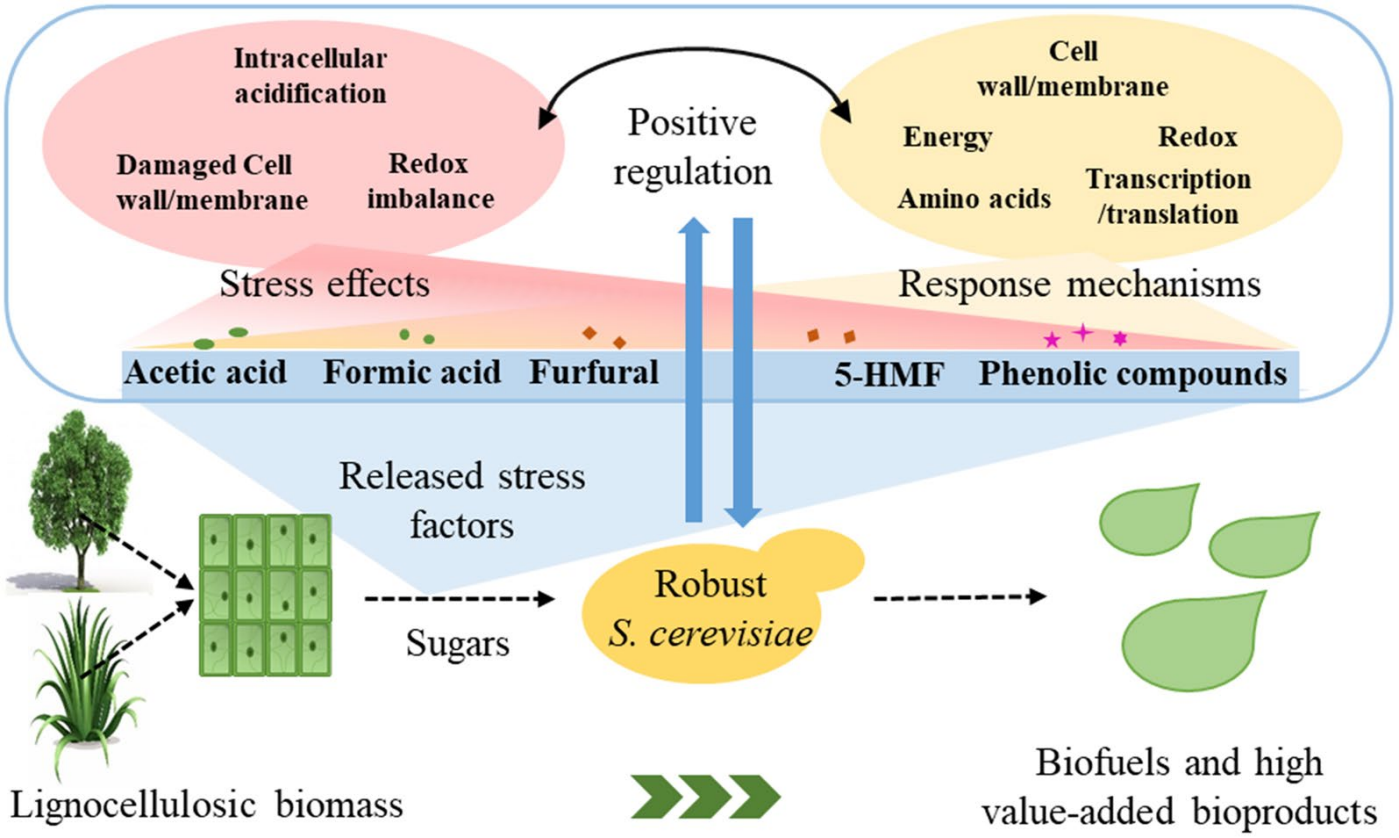

## Background

Carbon neutrality has attracted considerable attention recently due to the increasing CO_2_ levels in the atmosphere [1]. Biomass is one of the most abundant and renewable resource, which plays an important role to reduce the net CO_2_ emission [2, 3]. Lignocellulose is a major type of biomass with an estimated yield of hundreds of billions tonnes annually [4], which mainly includes herbaceous straws (e.g., crop straws [5], bagasse [5], leguminosae [6–8], solanaceae [9], sunflower [10], etc.), woody biomass (e.g., *paulowni*a *tomentosa* [11], poplar [12], etc., and various forestry residues), energy crops (e.g., miscanthus and switchgrass [13], etc.) and aquatic biomass. Compared with other biomass such as food crops (corn, soybeans, sugarcane, etc.) [14], and livestock and poultry dung [15]), lignocellulosic biomass has significant advantages like quick production, wide availability, low cost, and without interfering with food security [16]. It has been estimated that plenty of straws with total yield of 3165 million tonnes are produced as agricultural residues every year in the world [5]. Therefore, various technologies have been developed to convert lignocellulosic biomass to biofuels (e.g., bioethanol, butanol, CH_4_, H_2_, etc.) and high value-added products (e.g., sugars, alcohols, organic acids, terpenes, etc.) [13, 17].

**Fig. 1:**
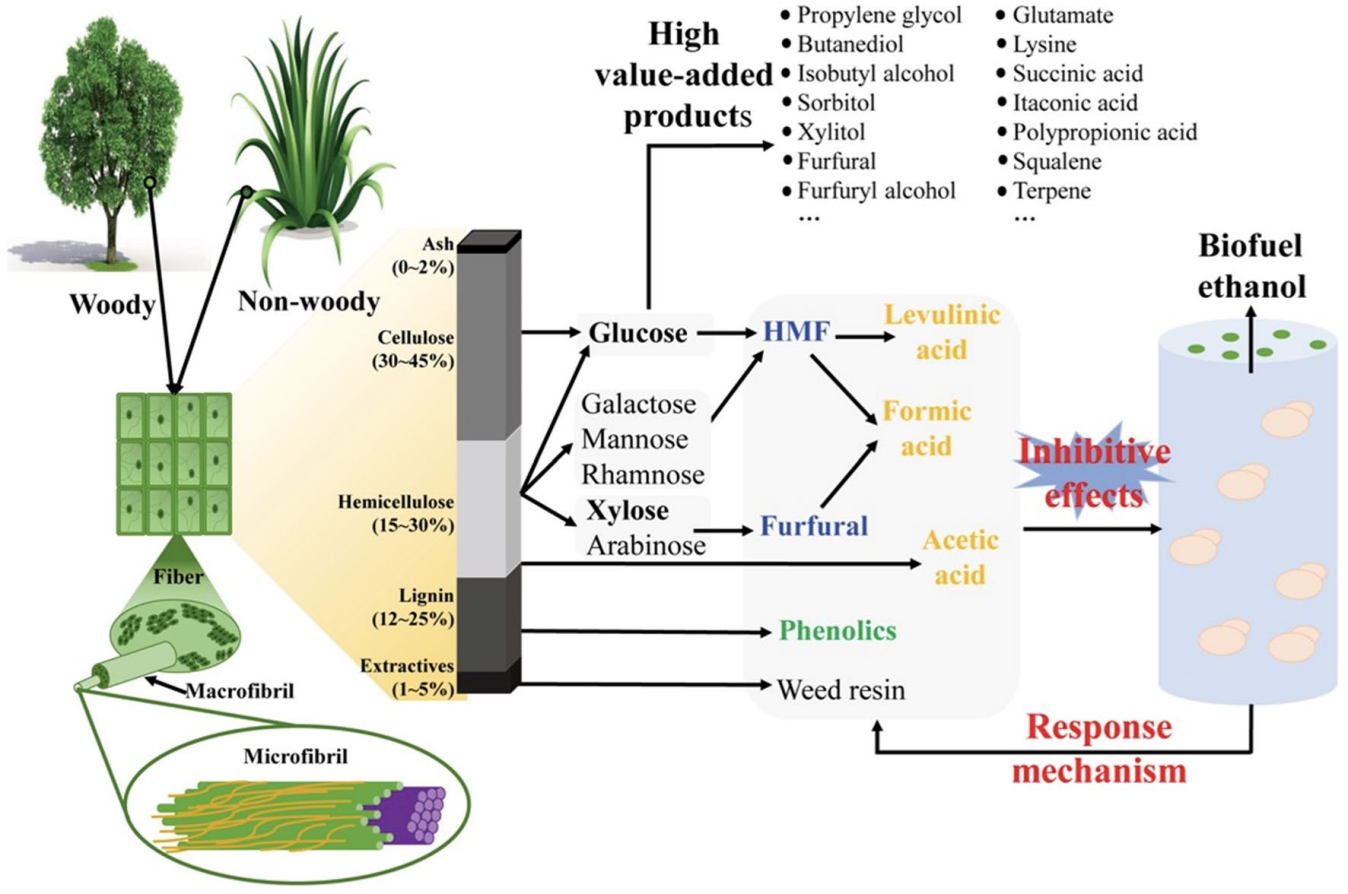
Main stress factors involved in production of lignocellulosic bioethanol by bioconversion

Lignocellulosic biomass is primarily composed of cellulose (25–55%), hemicellulose (8–50%) and lignin (10–35%) depending on the plant species [18]. Various fuels and chemicals can be produced by thermal, thermochemical and biological conversion of lignocellulosic biomass. However, for bioconversion, the complex structure constructed by the polymeric components necessitates pretreatment of the feedstock by various chemical, physical, and biological methods to deconstruct cell wall structure. Various chemical or combined pretreatments such as dilute acid, alkaline, steam explosion pretreatments, etc., have been developed to facilitate the conversion of carbohydrate polymers to monosaccharide (C5 and C6) [19, 20]. These monosaccharides can be further converted to various biofuels and high value-added platform chemicals such as ethanol, butanol, furfural, sorbitol, etc., through biorefinery technologies (Fig. 1) [13, 17]. However, despite that great efforts have been made to improve cellulose digestibility, cellulosic biofuels and chemicals have yet to be economically feasible at commercial scale [21].

Among the various biomass-derived biofuels, bioethanol is the one with commercialization and annual consumption reaching about 80 million tonnes. The second generation bioethanol with lignocellulose as the feedstock has been considered as one of the most promising way for bioethanol production because of no food-and-fuel debate [22]. The global annual production of cellulosic ethanol would be more than 400 million tonnes if the produced straws are converted to ethanol. However, the production of cellulosic ethanol is still facing various barriers on its way to industrialization. The inhomogeneity and complexity of biomass hydrolysate usually leads to significant decrease in sugar consumption rate and ethanol yield by *Saccharomyces cerevisiae*. Furthermore, the byproducts formed during pretreatment process, such as organic acids, furan aldehydes, and phenolic compounds, are strong inhibitors to *S. cerevisiae* growth and metabolism (Fig. 1 and Table 1) [23–25]. Thus, one of the obstacles for commercial production of cellulosic ethanol is the lack of robust strains that show strong tolerance to the stress factors [34]. Generally, several issues still need to be solved in order to construct more robust strains. First, most of the engineered strains have good resistance to single stress factor, but the tolerance ability to mixed stress factors are not strong enough [26, 31]. Second, the fermentation efficiency of the engineered strain is still low when lignocellulosic hydrolysate is used as fermentation medium [35, 36]. Third, the endogenous response and regulation mechanisms of *S. cerevisiae* to various stress factors still needs to be further analyzed and interpreted [37]. With the rapid development of modern biotechnology and synthetic biology, many works have been published for construction of robust strains and revealing the regulatory mechanism of *S. cerevisiae* in response to stress factors [38–42]. The response of *S. cerevisiae* has significant difference to various stress factors [24]. Although there are some similarities in different responsive metabolic pathways at the cellular level, the functional genes involved in can be much different [27, 43]. Considering the complexity of the metabolic regulation of *S. cerevisiae* and the certain gap for large-scale industrial applications, in this work, we have reviewed the stress response mechanisms of *S. cerevisiae* to various stress factors and the potential targeted genes. The strategies to improve strains’ tolerance ability also have been discussed, which may provide technical inspiration for construction of robust strains for industrial production of bioethanol with high efficiency.

**Table 1:** Recently reported fermentation performances of *S. cerevisiae* with lignocellulosic hydrolysate as the carbon source to produce ethanol

*S. cerevisiae*	Description	Feedstock	Initial sugar concentration	Stress factors	Ethanol conc. (g/L)	*Y*_E/S_ (g/g)	Refs.
TP1	*TFA7 PGK1*_*P*_-*PAD1*-*PGK1*_*T*_* ShBle ENO1*_*P*_-*ICT1*-*ENO1*_*T*_	2% synthetic media + 40% v/v concentrated hardwood spent sulphite liquor	Glucose 34.70 g/L xylose 92.70 g/L	Weak acids 15.70 g/L, furans 2.30 g/L, phenolics 2.00 g/L	12.20	0.26	[26]
s6H3T10	*UBI4*_*P*_*-HAA1-HAA1*_*T*_ *UBI4*_*P*_*-TYE7-TYE7*_*T*_	Corn stover	Glucose 93.88 g, xylose 14.81 g (Each kilogram of pretreated slurry)	Acetic acid 2.82 g, formic acid 1.53 g, furfural 0.21 g, 5-HMF 0.37 g, total phenols 2.33 g (Each kilogram of pretreated slurry)	47.50	0.44	[27]
MEC1133	PE-2, gre3::natMX4/gre3::kanMX4, pMEC149	*Paulownia elongata x fortunei*	Glucose < 5.00 g/L, xylose 55.80 g/L	Formic acid 0.71 g/L, acetic acid 5.67 g/L, levulinic acid 1.03 g/L, HMF 0.69 g/L, furfural 0.65 g/L, total phenols 8.25 g/L	14.20	0.33	[28]
XUSAE57	*BY4741*/xylA3*/*TAL1*/*XKS1*/*△gre3*/*△pho13*/evolved	Sugarcane bagasse	Glucose 26.20 g/L xylose 27.70 g/L	Acetic acid 2.50 g/L, phenolics 0.80 g/L	~ 23.00	0.49	[29]
PE-HAA1/PRS3	PE-2*ΔGRE3*, pMEC9003	*Paulownia tomentosa*	Glucose 30.00 g/L, xylose 11.30 g/L	Acetic acid 5.84 g/L, furfural 1.96 g/L, HMF 0.72 g/L	8.15	–	[11]
RED	Commercial *S. cerevisiae* (Fermentis)	Sugarcane bagasse	Glucose 18.8 g/L xylose 8.38 g/L	Formic acid 0.05 g/L, acetic acid 2.00 g/L, HMF 0.04 g/L, furfural 0.10 g/L, phenol 0.02 g/L, vanillin 0.13 g/L, acetovanillone 0.08 g/L	4.80	0.40	[30]
AR5	Tequila must (*Agave tequilana*)	Wheat straw	Glucose 14.52 g/L xylose 6.36 g/L	Acetic acid 1.78 g/L, HMF 0.57 g/L, furfural 0.25 g/L, vanillin 0.26 g/L	2.40	0.21	[30]
SXA-R2P-E	xylA*3/*TAL1*/*XKS1*/*Δgre3*/ *Δpho13*/evolved	Rice straw	Glucose 27.7 g/L xylose 20.20 g/L	Acetic acid, 1.00 g/L, phenolics 0.80 g/L, furfural 0.20 g/L	20.70	0.46	[31]
SXA-R2P-E	xylA*3/*TAL1*/*XKS1*/*Δgre3*/ *Δpho13*/evolved	Oak	Glucose 26.80 g/L xylose 16.00 g/L	Acetic acid 6.10 g/L, phenolics 1.30 g/L, furfural 0.60 g/L	17.70	0.43	[31]
MEC1133	PE-2, gre3::natMX4/gre3::kanMX4, pMEC149	Corn cob	Glucan 34.4% Xylan 29.0%	Acetic acid 4.20 g/L, furfural 2.40 g/L, HMF 0.20 g/L	25.50	0.47	[32]
TMB 3001	*XYL1*/*XYL2*/*XKS1*	Fresh bagasse H205	Total sugar 33.20 g/L	Acetic acid 4.00 g/L, formic acid 0.80 g/L, furfural 1.10 g/L, HMF 0.20 g/L, vanillin 4.10 g/L	8.80	0.26	[33]
TMB 3001	*XYL1*/*XYL2*/*XKS1*	Fresh bagasse H215	Total sugar 26.60 g/L	Acetic acid 4.50 g/L, formic acid 1.40 g/L, furfural 1.60 g/L, HMF 0.50 g/L, vanillin 4.50 g/L	6.00	0.22	[33]

## Response of *S. cerevisiae* to stress factors

Pretreatment is a pre-requisite step to facilitate the release of fermentable sugars either by chemical or enzymatic hydrolysis. Dilute acid pretreatment has been considered as one of the most promising approaches with potential commercial applications because the process employs cheap mineral acids such as sulfuric acid and can hydrolyze most of hemicelluloses to fermentable sugars [44]. However, stress factors including organic acids, furan aldehydes, and phenols are formed inevitably [45] (Fig. 1). The types and contents of these stress factors are closely related to the pretreatment conditions [46, 47]. Severe pretreatment conditions can promote the release of fermentable sugars, but also lead to formation of more stress factors. The yeast usually can give positive stress response to maintain its growth and reproduction in the presence of these stress factors [48]. However, there is a critical level of the stress factors concentration for yeast to initiate stress response. The growth and fermentation performance of the yeast can be greatly affected when the concentrations of the stress factors exceed the critical levels [23, 24].

### Organic acids

Organic acids stress factors mainly refer to formic acid and acetic acid. Acetic acid is mainly formed by deacetylation of the acetyl group of hemicelluloses, while formic acid can be formed by degradation of sugars [49]. The concentrations of organic acid stress factors in the hydrolysate are relatively higher than furans and phenols. The concentration of acetic acid is 1–15 g/L depending on the biomass feedstock and pretreatment methods used [50, 51]. The concentration of formic acid is usually lower than that of acetic acid, but the inhibitory effect of formic acid is stronger because of its lower pKa value (3.75 for formic acid in contrast to 4.75 of acetic acid). Intracellular acidification is the main reason for the inhibitive effects of organic acids on the cell growth [23, 24]. To maintain intracellular pH homeostasis, H^+^ is pumped out of the cell with the help of the ATPase in the membrane. A large amount of ATP is thus consumed with the exhaustion of H^+^, which leads to insufficient intracellular energy supply and affects cell growth and metabolism. At the same time, the extracellular organic acids could be transferred into cell. The intracellular organic acids continuously dissociate to release H^+^ for maintaining intracellular and extracellular ion balance. Since anions cannot be pumped out, the accumulated anions in the intracellular seriously affect the performance of cells [52].

### Furan aldehydes

Two types of furan aldehydes, namely furfural and 5-hydroxymethylfurfural (HMF), are usually detected in the hydrolysate obtained by thermochemical pretreatment of lignocellulosic biomass due to the dehydration reactions of pentoses and hexoses [53, 54]. Furfural concentrations usually ranges from 0.5 to 3 g/L, while the hydrolysate from corn stover can contain furfural as high as 11 g/L [55]. HMF usually found in spruce hydrolysate with the concentrations varied from 2.0 g/L to 5.9 g/L [56]. Intracellularly, furfural is reduced to furfuryl alcohol by alcohol dehydrogenases (ADH) or reductases (AKR/ARI) with NAD(P)H as a cofactor. Furfuryl alcohol could be rapidly converted by alcohol dehydrogenase [57, 58]. Furfural can also be oxidized to its acid form (furoic acid) by aldehyde oxidase, and then transformed into 2-oxoglutaric acid by a series enzyme, and further metabolized via tricarboxylic acid cycle (TCA) [24, 57]. The mechanism of intracellular detoxification of HMF is similar to that of furfural. However, due to the poorer ability of HMF to penetrate cell membrane than that of furfural, it takes longer time for the cells to detoxicate HMF [24, 59]. Furfural and HMF can be oxidized and reduced to lower-toxic substances in cells. However, this detoxification process breaks the intracellular redox balance. As a result, acetaldehyde accumulates during reduction of furfural/HMF, which is most likely to contribute to the inhibition of cell growth [23, 24].

Furan aldehydes have strong toxicity to *S. cerevisiae* [60]. The yeast showed a prolonged lag phase of 8 and 4 h for furfural- and HMF-treated cultures, respectively. The inhibition completely suppressed the cell growth at 120 mM of furfural or HMF independently. However, when both inhibitors were co-present in the media, cell growth was only recovered at 10 mM of each inhibitor, indicating that these inhibitors acted in negative synergic fashion even at low concentrations [61]. The activity of enzymes (hexokinase, triosephosphate dehydrogenase, and alcohol dehydrogenase) involved in glycolysis could be significantly inhibited under 10 mM furfural stress [62]. The genes related to oxidative stress response are upregulated in *S. cerevisiae* under furfural stress. Furfural can induce the accumulation of reactive oxygen species in cell, resulting in the irreversible damage to mitochondria and vacuole membrane, actin, cytoskeleton, and nuclear chromatin [63]. Furthermore, furfural and HMF can inhibit the protein and RNA synthesis, and reduce enzymatic and biological activities [61]. Therefore, although the concentrations of furan derivatives are usually lower than that of organic acids, they show more serious inhibitory effects on the growth and metabolism of yeasts.

### Phenolic compounds

Phenolic compounds present in the hydrolysates are typically formed by degradation of lignin, and usually exist in four forms, namely phenolic acids (e.g., ferulic acid), phenolic aldehydes (e.g., vanillin), phenolic ketones (e.g., 4-hydroxyacetophenone), and phenolic alcohols (e.g., homovanillyl alcohol) [64]. However, detailed characterization of the formed phenolic compounds is challenging because of their diversity and complexity in structure. The inhibition mechanism of these compounds might be attributed to cytoplasmic membrane invaginations, decreased membrane potential and permeability, repressed translation process, inhibited ribosomal function, and increased intracellular ROS concentration [65–68]. Phenolic compounds have serious stress effects on the growth and reproduction of *S. cerevisiae*, in which phenolic aldehydes shows the strongest toxicity. Coniferyl aldehyde even at a low concentration (1 mM) has been found to completely inhibit cell growth [69]. Phenolic compounds not only inhibit the cell growth, but also lead to deactivation of cellulases, resulting in the decrease in the yield of fermentable sugars by subsequent enzymatic hydrolysis [70, 71]. Therefore, improving the tolerance of strains to phenolic compounds is of great significance for bioethanol production from lignocellulose biomass.

### Synergetic effects of the stress factors

Various stress factors coexist in the hydrolysate, and thus the synergetic effects of these inhibitors exert more serious stress on *S. cerevisiae* [31]. Compared with single stress factor, mixed stress factors may induce more complex response with consumption of more intracellular energy resulting in a significant decrease in fermentation efficiency. However, the interactions among various stress factors, including positive and negative synergistic effects, still need further interpretation [72]. Chen et al. [43] reported that the glucose could be depleted in 60 h and 40 h under 2 g/L acetic acid and 1.5 g/L furfural stress, respectively, while the glucose was not exhausted even fermentation for 96 h under mixed acetic acid and furfural (2 + 1.5 g/L) stress. Under the stress effect of these factors, transmembrane transport processes play pivotal roles in response to acetic acid, and carbohydrate metabolic process is crucial for furfural stress. While the biological processes such as transmembrane transport, cellular amino acid metabolic process, and response to inhibitors are involved in *S. cerevisiae* resistance to the mixed fermentation inhibitors. Li et al. [41] revealed that more differentially expressed genes (DEGs) were involved in *S. cerevisiae* response to mixed acetic and formic acid stress compared with that to only acetic acid or formic acid stress. There were 294 unique DEGs among 657 total DEGs under the mixed acetic and formic acid stress, indicating that 45% of DEGs were unique in response to the mixed acid stress and 55% of DEGs were shared to respond single acid stress. It indicates that the strain adopts distinct regulatory mechanisms to reprogram cell metabolism in response to various stress factors.

Many studies have focused on revealing the stress response of *S. cerevisiae* to mixed stress factors with synthetic medium. However, when lignocellulose hydrolysate is used as fermentation medium, the stress response mechanisms of strains becomes more complicated which still need to be further interpreted [39, 73]. Revealing the stress response of *S. cerevisiae* induced by various stress factors is of great significance for developing robust yeasts for industrial purpose, since new strategies can be made by intensifying the key genes to detoxify the inhibitors.

## Response mechanisms of *S. cerevisiae* to stress factors

**Fig. 2:**
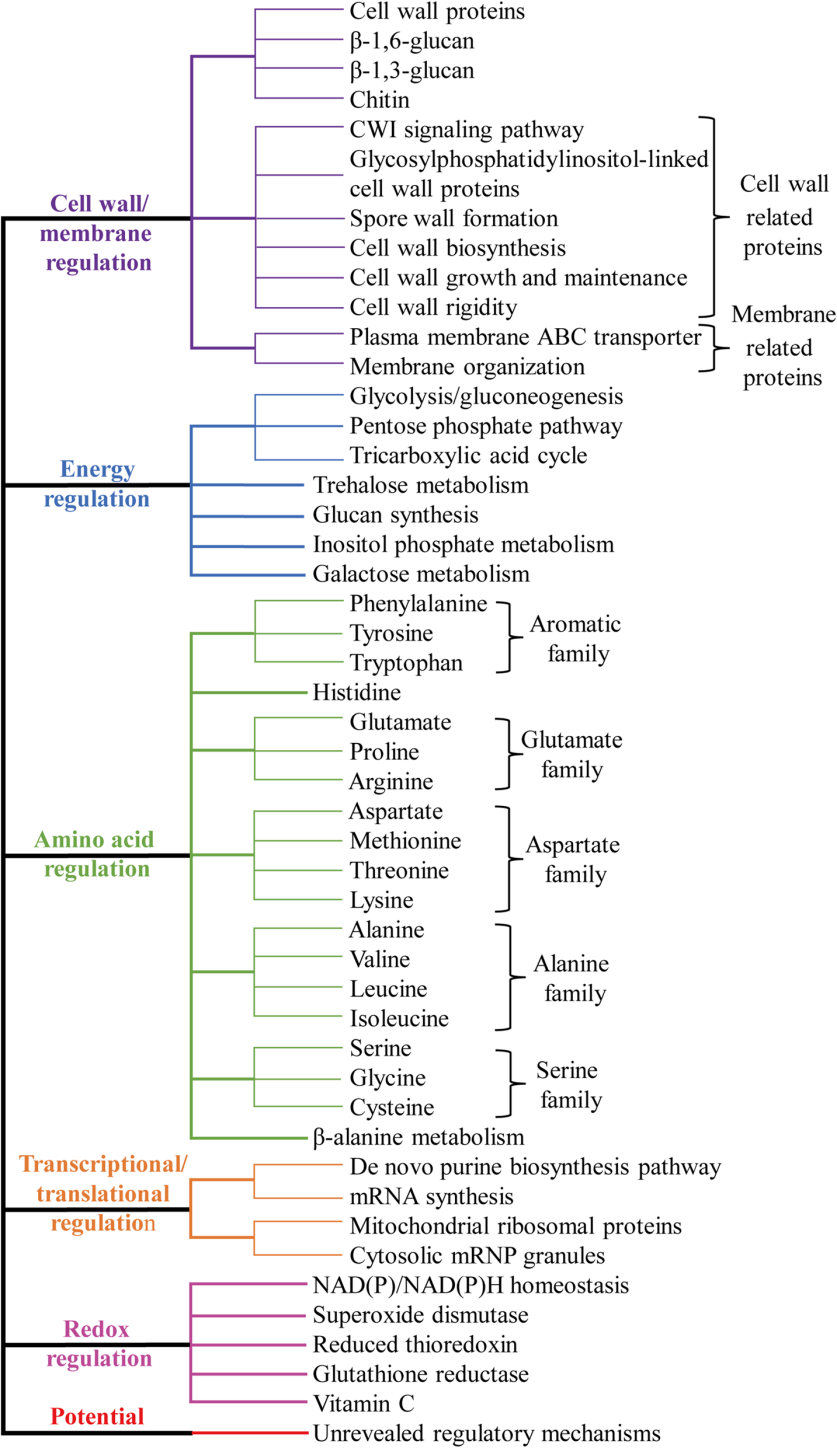
Endogenous regulation mechanisms of *S. cerevisiae* in response to stress factors

The response of *S. cerevisiae* to stress factors often presents global control at the cell level. With the continuous development of molecular biology and genetic engineering technology, many studies have focused on transcriptome and proteomic analysis to accurately and clearly reveal the endogenous regulation mechanisms of *S. cerevisiae* [42]. Based on these revealed results, the potential response mechanisms of *S. cerevisiae* have been summarized from cell wall/membrane regulation, energy regulation, amino acid regulation, transcriptional and translational regulation, and redox regulation, as shown in Fig. 2.

### Cell wall/membrane regulation

Cell wall/membrane is the first barrier of *S. cerevisiae* to resistant to stress factors [74–76]. The integrity and selective permeability of the cell wall/membrane are very important for strains to cope with external stress. The cell wall of *S. cerevisiae* contains four classes of macromolecules, namely cell wall proteins (CWPs), β-1,6-glucan, β-1,3-glucan, and chitin [77, 78]. These compositions and their degrees of cross-link can change in response to varied stress conditions [79, 80]. Cell wall is able to transmit signals to the cell membrane under stress, which remodels the components and structures of the membrane to adapt the corresponding stress [81]. The genes encoding these substances are increased to maintain the cell wall homeostasis in the presence of stress factors. The cell wall- and membrane-related genes, including *AUS1*, *COX14*, *GSC2*, *INP52, MCH2*, *MDG1*, *MDY2*, *NAB6*, *PEX3*, *PMT1*, *PSA1*, *SED1*, *SPT14*, *TIR4*, *USV1*, and *YTP1* have been found to upregulate in response to furfural and HMF stress [79]. Other genes that may not necessarily locate in cell wall but encode cell wall-related proteins are also involved in cell wall homeostasis in response to osmotic pressure, high temperature, furan aldehydes, or other stress factors, such as *CWP1*, *PIR3*, *PKC1*, *RHO1*, *ROM1*, *RLM1*, *SLT2*, and *YGP1*, related to cell wall integrity (CWI) signaling pathway; *PIR3*, *SPT14*, *SED1*, *SPI1*, and *TIR4* encode glycosylphosphatidylinositol-linked cell wall proteins; *DIT1*, *GIP1*, and *GSC2* for spore wall formation; *PSA1* and *USV1* for cell wall biosynthesis; *YPS3* for cell wall growth and maintenance; and *PMT1* for cell wall rigidity [82–84]. The genes involved in membrane function are also upregulated in the presence of stress, such as *AUS1*, *PDR15,* and *YOR1* involved in plasma membrane ATP-binding cassette (ABC) transporter, *HSP12* involved in maintaining membrane organization [76, 79]. These alteration results in cells that are more resistant to further cell wall degradation. Therefore, systemically regulating the expression of these genes to strengthen the composition and function of the cell barrier can effectively improve the stress resistance of strain.

### Energy regulation

Central carbon metabolism (CCM) is the main source of energy required for cell metabolism and provides precursors for other metabolites, which mainly includes glycolysis/gluconeogenesis, pentose phosphate pathway, and tricarboxylic acid cycle [85]. CCM has been found to play key roles in modulating yeast survival in response to stress factors. The genes involved in glycolysis are generally upregulated in response to 40 mM acetic acid [41], while the genes are downregulated when the acetic acid concentration reaches 300 mM [86]. Guo et al. [87] indicated that organic acids at moderate concentrations could stimulate the glycolytic flux, while higher acid level slowed down the glycolytic flux for both aerobic and anaerobic growth of *S. cerevisiae*. Chen et al*.* [43] found that carbohydrate metabolic process was crucial for strain response to furfural. The expression of most glycolytic enzymes are increased in industrial *S.* cere*visiae*, whereas those in the TCA cycle, glycogen and glycerol biosynthesis, and pentose phosphate pathway are largely downregulated in response to thermal stress [88].

Some other carbohydrates participating in CCM through oxidation–reduction reactions are also differentially expressed in response to stress factors. These substances play important roles in strain defense system. Trehalose metabolism (*TPS2*, *TSL1*, and *ATH1*) has been found to protect cell biomacromolecules from stress effects caused by acetic acid, high temperature, and high osmotic pressure [86, 89]. Glucan synthesis (*FKS1*, *FKS2*, and *ROM2*) has been found to involve in cell wall remodeling under acetic acid stress [90, 91]. The other carbohydrates, such as inositol phosphate and galactose, also play indelible roles in strains’ response to stress effects [41].

### Amino acid regulation

Amino acids are the key hallmarks and mediators for *S. cerevisiae* in response to stress factors, which essentially serve as a nitrogen source and the building blocks of proteins [92–94]. Increasing the expression of amino acid metabolism-related target genes or transcription factors (TFs) have been reported to contribute to the improving of the strain tolerance [27]. The genes involved in arginine, histidine, and tryptophan, were upregulated in response to acetic acid [86]. Tryptophan has a prominent contribution to maintaining cell membrane stability in *S. cerevisiae* [95], which is beneficial to enhance the tolerance of strain to stress factors. The genes related to the biosynthesis of cysteine and methionine (*CYS3* and *MET4*), histidine (*HIS4*), glycine (*GLY1*), and glutamate (*GDH1*) were identified as determinants of resistance to acetic acid [96]. Supplementation of cysteine, glycine, and glutamate (20 mg/L for each amino acid) could slightly increase *S. cerevisiae* resistance to acetic acid [96]. Except for these amino acids, alanine, aspartate, serine, threonine, proline, phenylalanine, tyrosine, valine, leucine, isoleucine, and beta-alanine metabolism are also involved in stress resistance [27, 97].

### Transcriptional and translational regulation

Genetic information flows from DNA to RNA, and then translates into proteins. This process is directly involved in the regulation of the growth and reproduction of strain, as well as in the anabolism and catabolism of key substances. *S. cerevisiae* must dynamically alter the levels of transcription and translation to respond the diverse stress [98]. The ADEnine requiring (ADE) genes, including *ADE1*, *ADE13,* and *ADE17*, etc., participate in the de novo purine biosynthesis pathway yielding inosine monophosphate (IMP) and adenosine 5′-monophosphate (AMP) [99]. Overexpressing of these ADE genes in *S. cerevisiae* have been found to enhance cell growth and ethanol productivity under mixed acetic acid, formic acid, furfural, and 5-HMF stress [100]. The genes involved in mRNA synthesis that directly affect transcriptional control and RNA process are downregulated in response to furfural, and the genes involved in mitochondrial ribosomal proteins are downregulated in response to acetic acid [86]. High concentrations of vanillin result in the repression of translational and the formation of cytosolic mRNP granules, leading to a reduction in overall protein synthesis levels and the limited translation of mRNAs [68, 101–103]. Regulating the expression of these genes to relieve stress-induced transcriptional and translational repression can be beneficial to enhance the tolerance of strain.

### Redox regulation

Redox regulation is a universal response of *S. cerevisiae* to resistant various stress factors. The oxidant defense systems mainly include NAD(P)^+^/NAD(P)H homeostasis, superoxide dismutase (SOD), reduced thioredoxin, glutathione reductase, and vitamin C [97, 104, 105]. NAD(P)/NAD(P)H homeostasis, correlated to NAD^+^ synthesis, and redox transformation from NAD(P)^+^ to NAD(P)H, is essential for preventing intracellular acidification induced by weak acid, and acidified phenolic or furan compound [97]. Various stress factors can induce the reactive oxygen species (ROS) accumulation in cell. SOD protects cells by scavenging ·O_2_^−^ (ROS). It has been reported that overexpression of *SET5* and *PPR1* or deletion of *ADY2* and *JJJ1* are beneficial to decrease the ROS accumulation and endow yeast increased tolerant ability to acetic acid [106, 107]. The nonenzymatic defense systems including reduced thioredoxin, glutathione reductase, and vitamin C, can act as reducing agents for scavenging free radicals to maintain intracellular redox homeostasis [108, 109].

## Target genes for improving *S. cerevisiae* tolerance to stress factors

Two states, activation and inhibition, are exhibited by yeast strains in response to stress factors, and ultimately are reflected by the upregulation or downregulation of genes. Based on the development of transcriptomics, proteomics, metabolomics, and other omics technologies, numerous potential targeted genes that have the positive contribution or regulatory function under stress conditions have been revealed through experiments (Tables 2 and 3). Due to the complexity of the endogenous regulatory mechanisms of strain and the lack of systematic understanding for the function of the targeted genes, the improvement of strain performance is very limited [141]. The genes and TFs that have been revealed to have contribution to improving the strain tolerance are summarized as shown in Tables 2 and 3.

**Table 2:** Reported target genes in improving *S. cerevisiae* resistance to stress factors

Target gene	Function	Location	Regulation	Stress factor	Refs.
*CCW12*	Cell wall mannoprotein	CWB	Overexpression	Acetic acid	[36]
*FPS1*	Aquaglyceroporin	CMB	Dephosphorylation	Acetic acid	[110]
*HOG1*	Mitogen-activated protein kinase	CMB	Activation/overexpression	Acetic acid and osmostress	[110, 111]
*ADY2*	Acetate transporter	CMB	Deletion	Acetic acid and ROS	[105]
*ATR1*	Multidrug efflux pump of the major facilitator superfamily	CMB	Overexpression	Coniferyl aldehyde, ferulic acid, and isoeugenol	[112]
*AZR1*	Plasma membrane transporter	CMB	Overexpression	Acetic acid	[113]
*FLR1*	Plasma membrane transporter of the major facilitator superfamily	CMB	Overexpression	Coniferyl aldehyde, ferulic acid, and isoeugenol	[112]
*PDR5*	Plasma membrane ATP-binding cassette (ABC) transporter	CMB	Overexpression	Vanillin	[40]
*YOR1*	Plasma membrane ATP-binding cassette (ABC) transporter	CMB	Overexpression	Vanillin	[40]
*SNQ2*	Plasma membrane ATP-binding cassette (ABC) transporter	CMB	Overexpression	Vanillin	[40]
*PMA1*	Plasma membrane P2-type H+-ATPase	CMB	Overexpression	Organic acids and ROS	[114]
*KAR2*	Endoplasmic reticulum chaperone BiP	CMB	Overexpression	Vanillin	[115]
*ACS2*	Acetyl-coA synthetase	BMM	Overexpression	Acetic acid	[116]
*ADE1/13/17*	ADEnine	BMM	Overexpression	Acetic acid	[100]
*BDH2*	Putative medium-chain alcohol dehydrogenase/reductases	BMM	Overexpression	Vanillin	[101]
*ADH1/6/7*	Alcohol dehydrogenase	BMM	Overexpression	Furfural, HMF, and vanillin	[117–119]
*ALD6/7*	Aldehyde dehydrogenase	BMM	Overexpression	Furfural and HMF	[120–122]
*GLR1*	Glutathione oxidoreductase	BMM	Overexpression	Furfural	[123]
*PAD1*	Phenylacrylic acid decarboxylase	BMM	Overexpression	Phenylacrylic acids	[124]
*PRS3*	Phosphoribosyl pyrophosphate synthetase	BMM	Overexpression	Acetic acid	[11, 39]
*PHO13*	*p*-Nitrophenylphosphatase	BMM	Deletion	Formic, acetic, levulinic acids, and furfural	[125, 126]
*RCK1*	Protein kinase	BMM	Overexpression	Acetic acid and oxidative stress	[38]
*SFA1*	Bifunctional alcohol dehydrogenase and formaldehyde dehydrogenase	BMM	Overexpression	Acetic acid	[35]
*WHI2*	Cytoplasmic globular scaffold protein	BMM	Overexpression	Acetic acid	[127]
*DBP2*	ATP-dependent RNA helicase of the DEAD-box protein family	BMM	Overexpression	Vanillin	[40]
*RPE1*	Ribulose 5-phosphate epimerase	BMM	Overexpression	Furfural	[128]
*TAL1*	Transaldolase	BMM	Overexpression	Furfural	[117, 125]
*TKL1*	Transketolase	BMM	Overexpression	Furfural	[128]
*GND1*	6-Phosphogluconate dehydrogenase	BMM/IRH	Overexpression	Furfural	[128]
*GSH1/2*	Gamma glutamylcysteine synthetase	BMM/IRH	Overexpression	Oxidative stress, furfural, and HMF	[129]
*IDP1*	Isocitrate dehydrogenase	BMM/IRH	Overexpression	Furfural	[123]
*ZWF1*	Glucose-6-phosphate dehydrogenase	BMM/IRH	Overexpression	Furfural	[123, 128]
*SET5*	Methyltransferase	IRH	Overexpression	Acetic acid and ROS	[106]
*JJJ1*	Co-chaperone that stimulates the ATPase activity of Ssa1p	IRH	Deletion	Acetic acid and ROS	[107]
*LacA*	Laccase	-	Heterologous expression	Vanillin	[115]

These genes are mainly involved in cell wall barrier (CWB), cell membrane barrier (CMB), basic metabolism maintenance (BMM), and intracellular redox homeostasis (IRH)

**Table 3:** Reported target TFs in improving *S. cerevisiae* resistance to stress factors

Target TF	Function	Regulation	Stress factor	Refs.
Ace2p	Activate transcription of genes encoding chitinases and glucanases	Overexpression	Acetic acid and furfural	[43]
Haa1p	Weak acid-responsive transcriptional activator	Overexpression	Organic acids	[130–132]
Hap4p	Transcriptional activator and global regulator of respiratory gene expression	Overexpression	Acetic acid, formic acid, and furfural	[27]
Msn2/4p	Stress-responsive transcriptional activator	Overexpression	Acetic acid, furfural, oxidative stress, and osmotic shock	[133–135]
Ppr1p	Zinc finger transcription factor	Overexpression	Acetic acid and ROS	[106]
Sfp1p	Regulates transcription of ribosomal protein and biogenesis genes	Overexpression	Acetic acid and furfural	[43]
Skn7p	Nuclear response regulator and transcription factor	Overexpression	Osmotic and oxidative	[136–138]
Tye7p	contribute to glycolytic genes activation	Overexpression	Acetic acid and furfural	[27]
Yap1p	Basic leucine zipper (bZIP) transcription factor	Overexpression	Oxidative stress, furfural, HMF, and vanillin	[67, 139, 140]
Yrr1p	Zn2-Cys6 zinc-finger transcription factor	Deletion	Vanillin	[40]

### Potential function genes involved in strain defense

**Fig. 3:**
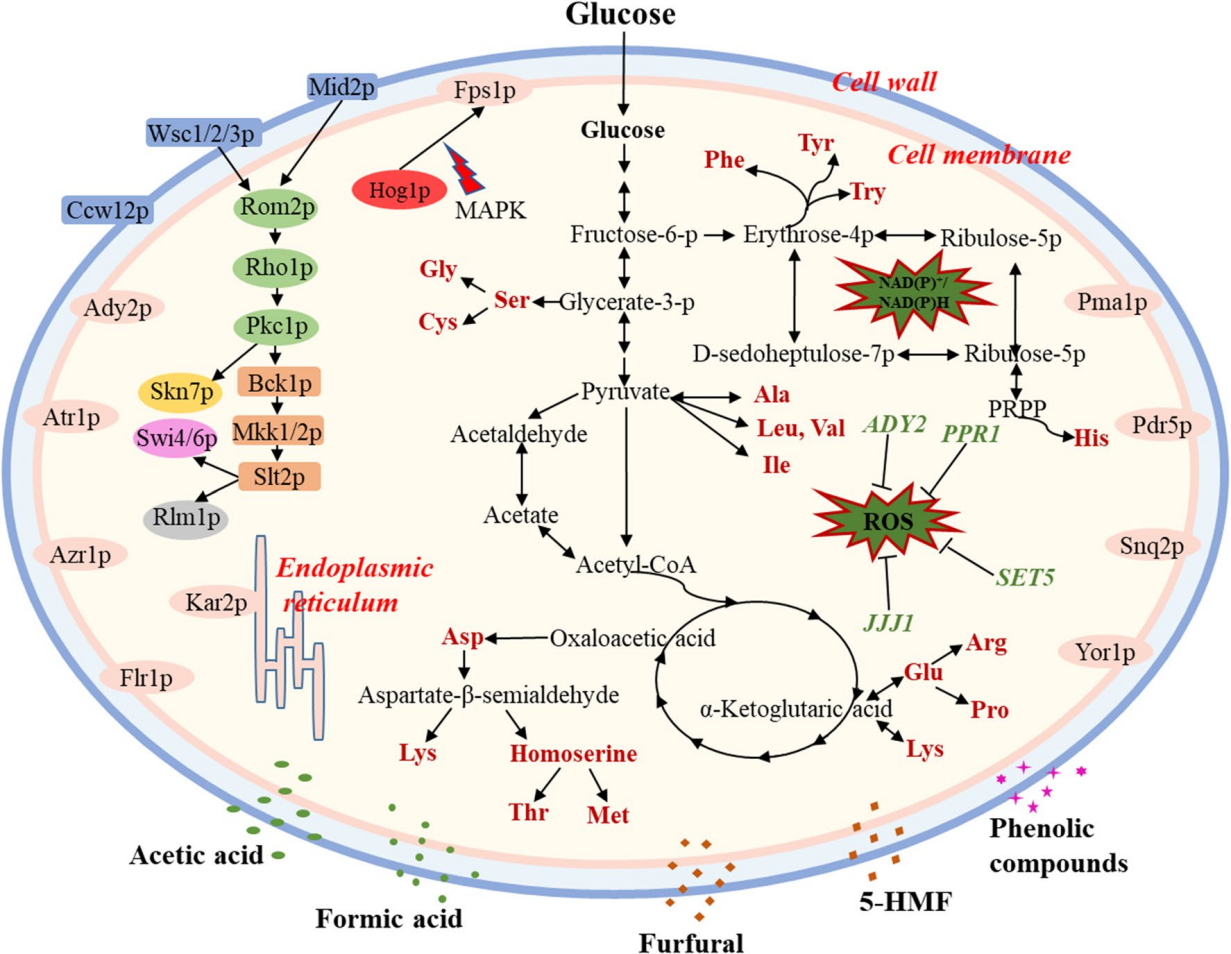
Schematic diagram of the multilevel defense of *S. cerevisiae* to different stress factors, including cell wall signals cascade, plasma membrane barrier, central carbon and amino acid metabolism, and intracellular redox homeostasis

The enzymes encoded by target genes is involved in the key signaling pathways and substance metabolism pathways, which contribute to the resistance ability of strains in response to specific stress. The experimentally proven functional genes are mainly involved in cell wall barrier (CWB), cell membrane barrier (CMB), basic metabolism maintenance (BMM), and intracellular redox homeostasis (IRH) (Table 2). These functional modules are closely correlated with the response mechanisms of *S. cerevisiae* (Fig. 2). When the cell wall receives stress signals, the protein Mid2p or Wsc1/2/3p (cell surface sensors) will be activated and then stimulate Rom2p (guanine nucleotide exchange factor) to activate the expression of Rho1p (small GTPase). The protein Rho1p that is involved in the establishment of cell polarity, regulates the expression of Pkc1p (protein kinase C). Pkc1p is essential for cell wall remodeling during growth, and in turn stimulates the cell wall integrity pathway. The PKC1-mediated signaling pathway further regulates the expression of Slt2p that is involved in the maintenance of cell wall integrity [74]. The activation of Slt2p results in changed transcription of more than twenty genes related to cell wall (Fig. 3). These genes cooperate to maintain the cell wall homeostasis [83]. This pattern of gene interaction is also presented in carbohydrate metabolism, amino acid metabolism, and intracellular redox regulation (Fig. 3 and Table 2). The key substances, glycerate-3-phosphate, pyruvate, oxaloacetic acid, ketoglutaric acid, and erythrose-4-phosphate, in central carbon metabolism are also involved in amino acid metabolism through enzymatic catalysis. Regulating the expression of the key genes in pentose phosphate pathway (*TAL1, TKL1, GND1*, and *ZWF1*) not only regulates energy metabolism and basic substance metabolism, but also helps maintain intracellular NADP^+^/NADPH content that participates in the intracellular redox regulation [117, 125, 128].

This complex regulatory network is related to the fact that the improvement of the strain performance depending on single gene positive regulation is not enough to significantly improve the fermentation efficiency in the presence of stress factors. The up-/down-regulation of the key genes results in the accumulation or deprivation of the downstream/upstream products, which in turn leads to the disequilibrium of metabolic networks (such as feedback regulation). Therefore, assisted domestication or mutagenesis may be a method to balance cell metabolic homeostasis on the basis of limited improved strain performance by directional modification [142].

### Potential transcription factors involved in strain defense

TFs can regulate a series of genes. The disturbance caused by the up-/down-regulation of a single TF may be more significant than that caused by a single gene. However, the performance of the strain obtained by directional modification of single TF still cannot reach the ideal level when lignocellulosic hydrolysate is used as fermentation medium to produce ethanol [31, 143]. This is not only related to the complex fermentation environment of hydrolysate, but also related to the unclear regulatory mechanism of TFs. Therefore, the regulation mechanisms of the typical TFs are briefly summarized (Table 3).

### Haa1p regulation

Haa1p is involved in the endogenous regulation of *S. cerevisiae* and the reduction of intracellular acetate concentration under acetic acid stress [144]. Except to acetic acid, Haa1p is also related with yeast resistance to lactic acid and propionic acid [131, 145]. Haa1p rapidly relocates from the cytoplasm to the nucleus in the presence of acetic acid or lactic acid [146, 147]. The DNA binding of Haa1p can be induced by acetic acid. Acetate binds to the N-terminal 150-residue region (N-terminal Zn-binding domain), and the transcriptional activation domain is located between amino acid residues 230 and 483 [148]. Haa1p involved in adaptation to weak acid stress by inducing the transcription of genes *TPO2* and *TPO3*, which localize on the plasma membrane and encodes polyamine transporter of the major facilitator superfamily [131, 149]. Under the acetic acid stress, Haa1p also regulates the genes that encode protein kinases, multidrug resistance transporters, membrane transporter, membrane stress proteins [144, 150]. The genes involved in lipid metabolism, and nucleic acid processing are also directly or indirectly regulated by Haa1p in response to acetic acid [144]. The strain overexpressed *HAA1* exhibits improved sugar consumption and ethanol production from glucose or xylose in the presence of acetic acid [11, 151]. These conclusions can prove that Haa1p has a prominent contribution to improving the tolerance of *S. cerevisiae* to weak acid in hydrolysates, especially acetic acid.

### Msn2/4p regulation

Msn2p and Msn4p are two homologous stress-responsive TFs involved in *S. cerevisiae* transcriptional response to environmental stress response, such as acetic acid, furfural, oxidative stress, osmotic shock, glucose starvation, high ethanol concentrations, and high temperature [92, 152]. Once *S. cerevisiae* is challenged by these stress factors, Msn2/4 are rapidly dephosphorylated and translocated into the nucleus [153, 154]. Msn2/4p binds DNA at stress response elements of responsive genes and activates hundreds of stress-related genes as a consequence to various stress conditions [155]. The reported functional domains of *MSN2* include the C-terminal zinc finger DNA-binding domain (DBD), the nuclear localization signal (NLS) region, the nuclear export signal (NES) region, and the imperative transcriptional activating domain (TAD) at the N-terminus [154, 156–158]. The genes encoding antioxidant enzymes (*CTT1*, *SOD1*, *SOD2*, *PRX1*, and *TSA2*) were regulated by *MSN2*/*4*, which is beneficial for removing reactive oxygen species (ROS) to eliminate stress from various stress factors [104, 135]. The stress-related genes regulated by *MSN2*/*4* also involved in protein quality control (*HSP12*, *HSP26*, *HSP42*, *HSP82, HSP104*, *SSA1*, and *SSA4*), mitochondrial respiratory (*COX5b*, *COX17*, and *COX20*), glycogen synthetic (*GSY1*, *GSY2*, and *GLC3*), and pentose phosphate pathway (*SOL4*, *GND2*, and *TKL2*) [135, 159, 160]. Previous studies have indicated that the transcription abundance of *MSN2/4* could be significantly increased in response to acetic acid, furfural, or high temperature, etc. [79, 134]. These facts demonstrate that overexpression of *MSN2/4* is a promising approach for constructing robust strains with improved tolerance and fermentation performance.

### Yap1p regulation

The basic leucine-zipper transcription factor Yap1p transits from the cytoplasm to the nucleus when triggered by oxidative stress, and is degraded in the nucleus after the oxidative stress has removed [143]. Cysteine-rich domain is the active site of Yap1p [137]. The specific activity of enzymes involved in oxidative detoxification, such as glucose-6-phosphate dehydrogenase, superoxide dismutase, and glutathione reductase could be decreased with the knockout of Yap1p, which increases the sensitivity of yeast cell to hydrogen peroxide and chemicals that generate superoxide anion radicals [161, 162]. In addition to oxidative stress, furfural and HMF acting as thiol-reactive electrophiles could directly activate Yap1p. Overexpression of *YAP1* enhances the tolerance of *S. cerevisiae* to furfural and HMF by activating catalase expression (*CTA1* and *CTT1*) [120, 140, 163].

Based on omics analysis and various molecular techniques, more tolerant-related TFs and their regulatory mechanisms are being revealed, providing biological basis for robust strain construction with rational design. Haa1p, Msn2/4p, and Yap1p are typical TFs in improving the tolerance of *S. cerevisiae*. Other TFs, such as Skn7p (involved in oxidative stress response) [137], Sfp1p and Ace2p (involved in acetic acid and furfural stress response) [43] are also involved in the stress response of *S. cerevisiae*. In addition to these TFs verified by genetic engineering, transcriptome and other omics analysis also have revealed some potential TFs that contribute to stress response, such as Hcm1p, Fkh1/2p, Pdr1/3p, Met4p, etc. [52, 120, 164]. Tolerance-related TFs are synergistically involved in strain stress response. Current experimental results have confirmed that the performance of the engineered strains can be effectively improved by regulating key genes or TFs in the laboratory stage, but the stress resistance of the strains still needs to be improved in industrial applications [26]. It once again suggests that the improvement of strain robustness should be based on synergetic regulation of multi-genes/pathways. Therefore, understanding the response mechanism of *S. cerevisiae* to various stress factors and the key genes or TFs, is of great significance for constructing robust strains.

## Strategies for construction of robust strains

**Fig. 4:**
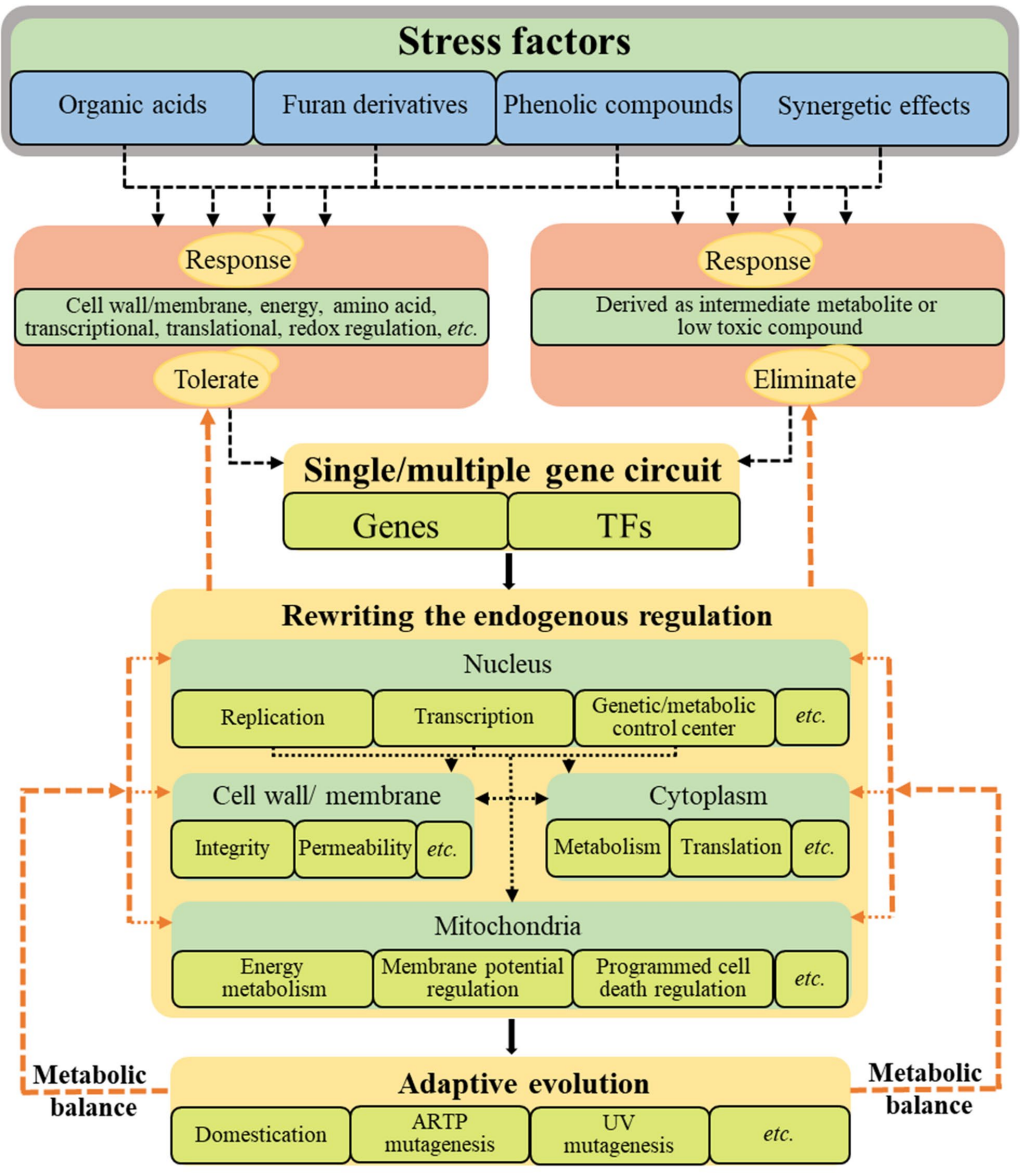
Possible strategies to construct robust *S. cerevisiae*. The response performance of *S. cerevisiae* to stress factors can be improved with the construction of the tolerant and eliminated pathway in cell. The expression of the single/multiple genes circuit induces the rewriting of the endogenous regulation of *S. cerevisiae*. Assisting in adaptive evolution to balance the metabolic load of strain can further promote the improvement of *S. cerevisiae* robustness

The interaction of multi-genes/pathways for stress-induced response and the endogenous regulation mechanisms of *S. cerevisiae* on stress factors are schematically shown in Figs. 2 and 3. The complex endogenous regulatory mechanisms of the yeast, as well as the fermentation performance and tolerability of the engineered strains suggest that the positive regulation of a single gene may be unable to cope with the cell-level disturbances. The mixed stress factors in the fermentation system puts forward higher requirement for the robustness of the yeast. Therefore, synergetic regulation of multigene to establish multi-tolerant system may be one of the most efficient methods to improve the fermentation performance of the yeasts in response to stress factors (Fig. 4). As a result, researchers have proposed to build a robust system based on genetic engineering (such as, synthetic chromosome rearrangement and modification by *LoxP*-mediated evolution (SCRaMbLE) [165–167], global transcription machinery engineering (gTME) [168], RNA interference (RNAi)-assisted genome evolution (RAGE) [169], automated multiplex genome-scale engineering (CRISPR–Cas) [170]), which can induce the rewriting of the endogenous regulation to establish tolerance response. At the same time, domestication and mutagenesis (such as, atmospheric and room temperature plasma (ARTP) and UV mutagenesis) is assisted to balance the metabolic load of strain [171–173]. Furthermore, targeted elimination of stress factors such as weak acids, furans, and phenols, has been proposed to cope with the stress from hydrolysate components, considering that these substances can be converted to less toxic substances by specific enzymes [50, 57]. This strategy also relies on the homologous or heterologous expression of the target genes. Assisting adaptive evolution will be more conducive to the target strain construction with the disturbance of the endogenous regulation caused by the target gene expression.

### Single-site modification

At present, many works have been done to endow *S. cerevisiae* with tolerance to certain stress factors through directional modification of the up-/down-regulation of the key genes (Tables 2 and 3). However, this strategy just can increase the strain robustness with limited improvement. A common problem has been encountered is that the engineered yeasts still show relatively low fermentation efficiency when lignocellulose hydrolysate is used as the carbon source (Table 1). This is because the co-presence of various stress factors in hydrolysate causing more serious stress on the growth of the yeast.

### Multi-site modification

Multi-site modification based on rational and irrational genetic editing techniques may be one of the effective approaches to obtain robust strains [174]. Rational construction mainly relies on the directional design of functional genes to construct multiple defense system. Irrational construction can be realized through domestication, ARTP, and other technical means to achieve non-directional change of multiple loci, so as to establish a mutant yeast library, and obtain excellent strains through screening and evaluation. Xu et al. [142] developed a multilevel defense system (MDS) by random assembly of tolerance genetic circuits, adaptive evolution (e.g., ARTP), and multi-step screening to obtain industrial yeasts with higher robustness and productivity. The strain integrated with MDS can tolerant multiple stress such as high surge, high temperature, and ethanol. The fermentation performance was significantly improved compared with single defense system. Si et al*.* [169] reported the RAGE technology as a generally applicable method for genome-scale engineering in *S. cerevisiae.* RNAi-assisted genome evolution could improve the acetic acid tolerance of strain. Except for the application of RAGE, Si et al. [170] developed a platform for automated multiplex genome-scale engineering in *S. cerevisiae*, which iteratively integrated the functional gene fragment of cDNA library into the genome with the aid of CRISPR–Cas. This system allowed functional mapping and multiplex optimization on a genome scale for diverse phenotypes, such as acetic acid tolerance.

Multilevel directional modification can be obtained by integration of multiple target genes/fragments with specific functions, in which designing and revealing the target genes/fragments with superior performance is the basis for successful construction of robust strain. Omics data analysis have indicated that cell wall/membrane integrity, energy metabolism, amino acid metabolism (protein quality control), and redox homeostasis play an important role in strain resistance (Figs. 2 and 3). Therefore, it is expected to construct robust *S. cerevisiae* strain by regulating the expression of these genes. Meanwhile, assisting domestication, mutagenesis, and other techniques to adjust the degree of adaptation between the genetic circuits and chassis, can further improve the robustness of the strain.

### Metabolism and elimination of stress factors

Improving the ability of strain to eliminate stress factors (e.g., organic acids, furan aldehydes, and phenols) is another tolerant feature of the robust *S. cerevisiae*. The up-take of these stress factors enables in situ detoxification of lignocellulosic hydrolyzates to lessen their inhibitory effects. Weak acids enter yeast cells by free diffusion and active transportation [175]. By combining a nicotinamide adenine dinucleotide (NADH)-consuming acetate consumption pathway and an NADH-producing xylose utilization pathway, Wei et al. [50] successfully constructed a pathway to convert toxic acetic acid to ethanol in engineered *S. cerevisiae* under anaerobic conditions. Furfural and HMF can be used as the sole carbon source for cell growth by *Amorphotheca resinae *ZN1, and converted to low-toxic compounds for *S. cerevisiae* [57, 122]. Overexpression of dehydrogenases (*ADH6*/*7*) and pentose phosphate pathway (*ZWF1*) genes can increase the reduction capacity of *S. cerevisiae* to furfural and 5-HMF [119, 122, 123]. For the elimination of phenolic compounds, the reported studies mainly rely on laccase to degrade it into low-toxic compounds outside the cells. The growth rate and ethanol productivity of *S. cerevisiae* were increased with the laccase treatment for the phenolic compounds [176]. Lei et al. [115] integrated laccase gene (lacA, from *Trametes *sp. AH28-2) that fused with *a*-factor signal sequence into *S. cerevisiae* CEN.PK, and further overexpressed chaperone gene (*KAR2*) to promote the translocation of laccase. Their results showed that vanillin-specific conversion rate was increased, and the strain tolerance to vanillin was increased.

The above-mentioned construction strategies can be achieved by the upregulation, downregulation, or knockout of the target genes and TFs. Therefore, systematic understanding the endogenous regulatory mechanisms of strain can lay a foundation for robust strain construction for efficiently conversion of lignocellulosic hydrolysate to biofuels and chemicals.

## Conclusions and prospects

Bioconversion of lignocellulosic biomass to bioethanol is one of the important ways to achieve carbon neutrality. However, relatively low tolerance of *S. cerevisiae* to the stress factors in the hydrolysate is one of the key obstacles for direct utilization of lignocellulosic hydrolysate. *S. cerevisiae* displays somewhat different response to different stress factors, such as organic acids that mainly cause intracellular acidification, furan aldehydes that induce intracellular redox imbalance, and phenolic compounds that destroy the cell membrane integrity. These negative effects can induce the rewriting of the endogenous regulatory of *S. cerevisiae* by differential expression of a series of genes mainly regarding the cell wall/membrane, energy, amino acids, transcriptional and translational, and redox regulation. Based on the above response mechanism, the robustness of *S. cerevisiae* might be improved by designing target genetic circuit according to corresponding regulatory points. Due to the complex regulatory network of *S. cerevisiae*, simple regulation of several genes or TFs usually cannot meet the requirements to obtain enough high robustness of strains for industrial application. Many strategies including single-site modification, multi-site modification, and metabolic intensification to improve strain tolerance and eliminate stress factors have been developed for construction of robust strains owing to the rapid development of modern biotechnology and synthetic biology such as SCRaMbLE, RAGE, gTME, CRISPR–Cas, adaptive evolution, multi-step screening, etc. With the aim of further increasing the tolerance of *S. cerevisiae*, various strategies can be employed by effective integration of the target genetic circuit. Therefore, future works can be done at least from following aspects.

(a) Diverse gene circuits for improving the strain robustness could be designed, including cell barriers, energy supply pathways, antioxidant defense, identification, and elimination systems. Combined gene circuits may be obtained by integrating functional modules with better tolerance.

(b) Construction of engineered cells with different tolerance preferences using the same parent strain, respectively, to reduce the metabolic burden of multigene integration. These cells then can be co-cultured to eliminate various stress factors in hydrolysate. Therefore, the concept of “functional flora” can be applied to cope with the complex stress of multi-stress factors by using several independent cells (the same parent strain) with different functions.

(c) Combining directional and non-directional modification techniques to balance the metabolic burden of the robust strains to tolerant multi-stress factors. The strain performance can be improved by target genetic modification. By assistance of domestication, ARTP, and other mutagenesis technologies, it is expected to adjust the metabolic load of strains and further improve the performance.

## Data Availability

Not applicable.

## Abbreviations

ABC: ATP-binding cassette
ADH: Furfuryl alcohol dehydrogenases
AMP: Adenosine 5′-monophosphate
ARTP: Atmospheric and room temperature plasma
BMM: Basic metabolism maintenance
CCM: Central carbon metabolism
CMB: Cell membrane barrier
CWB: Cell wall barrier
CWI: Cell wall integrity
CWP: Cell wall protein
DBD: DNA-binding domain
DEG: Differentially expressed genes
gTME: Global transcription machinery engineering
HMF: 5-Hydroxymethylfurfural
IMP: Inosine monophosphate
IRH: Intracellular redox homeostasis
MDS: Multilevel defense system
NES: Nuclear export signal
NLS: Nuclear localization signal
RAGE: RNA interference (RNAi)-assisted genome evolution
ROS: Reactive oxygen species
SOD: Superoxide dismutase
TAD: Transcriptional activating domain
TCA: Tricarboxylic acid cycle
